# Afghanistan's humanitarian crisis and its impacts on the mental health of healthcare workers during COVID-19

**DOI:** 10.1017/gmh.2022.3

**Published:** 2022

**Authors:** Zarmina Islam, Aiman Rija, Parvathy Mohanan, Khulud Qamar, Kainat Jahangir, Faisal A. Nawaz, Mohammad Yasir Essar

**Affiliations:** 1 Faculty of Medicine, Dow Medical College, Dow University of Health Sciences, Karachi, Pakistan; 2 Faculty of Medicine, Medical University Sofia, Sofia, Bulgaria; 3 College of Medicine, Mohammed Bin Rashid University of Medicine and Health Sciences, Dubai, United Arab Emirates; 4 Faculty of Dentistry, Kabul University of Medical Sciences, Kabul, Afghanistan

**Keywords:** Afghanistan, burnout, coronavirus, economic crisis, health care workers, humanitarian crisis, mental health

## Abstract

Afghanistan's humanitarian crisis has severely impacted the mental health of frontline workers. With the introduction of the Taliban government, ongoing civil unrest, and other forms of violent attacks, healthcare workers (HCWs) continue to provide patient care despite minimal resources. A severe contraction in the economy, poor supply of medications, political turmoil, and insufficient humanitarian aid have added to pre-existing problems. High levels of insecurity and instability as well as decades of traumatic experiences have contributed to increasing mental health challenges amongst frontline workers. Despite the scarcity of mental health services, HCWs continue to persevere with their service to the community. However, inadequate interventions may have serious implications for HCWs bearing the brunt of multiple traumas. Thus, governmental and international involvement is needed to address both the economic and psychological needs of HCWs in Afghanistan.

## Introduction

The ‘War on Terror’ is estimated to cost more than US$ 2 trillion, impacting over 40 000 civilian lives in Afghanistan (Knickmeyer, 2021; Reality Check Team BBC, 2021). The increasing number of civilian casualties and worsening national insecurities are major factors that influence this current state of crisis. However, despite such loss, international relief efforts amidst this humanitarian crisis continue to be insufficient for healthcare workers (HCWs), being the most vulnerable targets of violence and mental health issues in Afghanistan (Lucero-Prisno III *et al*., 2020*a*, 2020*b*; Essar *et al*., 2021; Leahy, 2021).

Afghanistan's humanitarian crisis involves multiple converging systems which makes it particularly challenging during a pandemic to address widening gaps in mental health needs (Essar *et al*., 2021). Among these are inflation, social and civil unrest, reduced healthcare capacity, infrastructural damage, rampant Coronavirus Disease 2019 (COVID-19) delta variant, and violent attacks, collectively resulting in high levels of stress in the general population (Essar *et al*., 2021). As of 11 January 2022, over 158 452 COVID-19 cases and 7374 deaths have been registered (Worldometer, 2022).

Moreover, questions on international relations with the Taliban government have created ambiguity for security and stability in the country. Conversely, the provision of women's rights has been a mainstream concern with ongoing protests. These interdependent factors are impairing the implementation of public health measures, thus creating a vacuum for conflict, instability, and burden on the healthcare system (Essar *et al*., 2021). Consequently, HCWs are in a vulnerable position. This is evident by the gender gap in the workforce with female HCWs representing merely 22% of doctors and 21% of nurses in Afghanistan (International Organization for Migration, 2020). Additionally, the United Nations Assistance Mission in Afghanistan (UNAMA) reported 75 violent incidents in 2019 against HCWs in comparison to 65 in 2018 (Lucero-Prisno III *et al*., 2020*a*, 2020*b*). The current national turmoil has collectively increased the workload on frontline workers resulting in rising mental health concerns (Leahy, 2021).

Since 1978, repeated cycles of war coupled with the impacts of poverty and displacement have contributed to depression, anxiety, and post-traumatic stress disorder (PTSD) in half the population of Afghanistan (Pedneault, 2019). Subsequently, a fragile health framework that is ill-equipped to provide mental healthcare predisposes this nation to a large mental health crisis (Kovess-Masfety *et al*., 2021*a*, 2021*b*). Husna Safi, an Afghan Canadian psychotherapist, states that generations of Afghans have been born amid the struggle and many have never known peace, detailing the role of intergenerational trauma (Kovess-Masfety *et al*., 2021*a*, 2021*b*). A recent nationwide study including 4445 citizens found a high portion of exposure to trauma is evident in Afghans, with 86% of respondents either having personally experienced or seen at least one traumatic occasion in their lives. Moreover, 47% of the population have experienced mental distress with women reporting higher levels of distress. The PTSD predominance was comparable to what was detailed in nations that have experienced war (Islam *et al*., 2021*b*). Astoundingly, the study mentioned that PTSD cases were not as recognizable to therapists in Afghanistan. This may be because traumatic experiences have been normalized and hence do not incite any mental health consultations, which is an important consideration for HCWs dealing with mental health issues (Kovess-Masfety *et al*., 2021*a*, 2021*b*). With <1% of medical training allocated to mental health, 0.5 mental health facilities per 100 000 people, and only 320 hospital beds available for mental health patients, major roadblocks in access to mental health services continue to persist (World Health Organization, Health and Ministry of Public Health Afghanistan, 2006). Thus, this paper aims to comment on the ever-increasing mental health concerns for HCWs in Afghanistan and provide scalable recommendations.

## Discussion

Challenges in maintaining a stable healthcare infrastructure have chronically plagued Afghanistan. This is seen by the ratio of nine HCWs and two physicians per 10 000 people in the region (Lucero-Prisno III *et al*., 2020*a*, 2020*b*). Furthermore, access to healthcare services is aggravated by existing respiratory health issues (Essar *et al*., 2021; Shah *et al*., 2021). Failure to document cases and halt the spread of the delta variant is exposing HCWs to constant physical and psychological risks (International Organization for Migration, 2020; Essar *et al*., 2021). Extended working hours, insufficient funding, suboptimal preparedness, and multiple traumatic experiences have worsened the pre-existing mental health struggles among HCWs (International Organization for Migration, 2020; Leahy, 2021). Moreover, lack of adequate gear, supplies, or preparation during the pandemic has added to this crisis. According to the Ministry of Public Health in Afghanistan, HCWs are bearing the brunt of uncontrolled transmission with 10% of all affirmed cases among healthcare staff, and 53 workers have died as a result of this catastrophe (International Organization for Migration, 2020). Afghanistan's remarkable advances in public health celebrated over the past two decades were vastly conceivable due to the efforts of women, including doctors, nurses, midwives, and community health laborers. Four out of 100 extraordinary nurses and midwives leaders over the world in 2020 have been recognized from Afghanistan (Parray *et al*., 2021). At the same time, female HCWs continue to struggle between the expanding health needs of the population amidst ongoing political conflict (Parray *et al*., 2021).

**Fig. 1.**
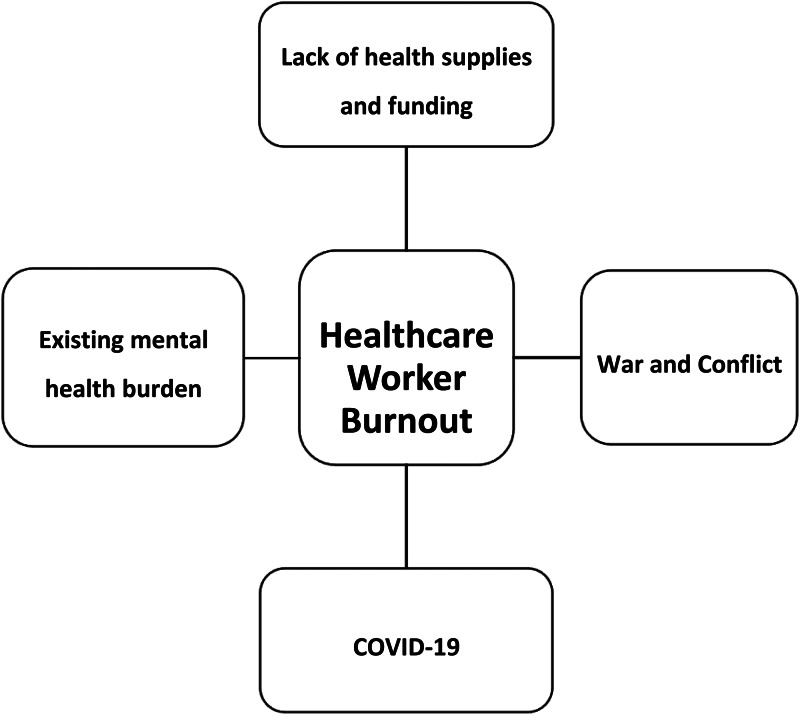
Contributing factors to healthcare worker burnout in Afghanistan.

Among this, the impact of war and consequences of the pandemic are primary negative contributors to the frontline workforce, according to a study done in 2020 (Lucero-Prisno III *et al*., 2020*a*, 2020*b*). Chronic funding issues have led to the suspension of salaries resulting in financial distress (Nemat *et al*., 2021). Moreover, the limited number of functioning hospitals, shortage of active working staff, and restricted humanitarian aid exacerbate such working conditions (Essar *et al*., 2021; Nemat *et al*., 2021). Afghanistan's political circumstances have halted the availability of female literacy for years, resulting in the lesser numbers of female HCWs. Furthermore, emerging conflicts have caused additional problems for female HCWs including travel, access to resources, and continued professional development. Moreover, in a place where it is customary for a female patient to be seen by a female HCW, access to women's health needs has become extremely scarce (Hashemi, 2012; Ghebreyesus and Al-Mandhari, 2021). Ultimately, this has led to poor outcomes in the performance of the health system, and loss of trust arising from increasing violence which is reported to affect over 15% of HCWs (Essar *et al*., 2021). Multifactorial causes, such as mass displacements and immigration needs, losing loved ones, and bearing the emotional needs of patients, continue to impact workplace efficacy (Kovess-Masfety *et al*., 2021*a*, 2021*b*). Whilst dealing with an increase of patient inflow and emotional needs, there happens to be no psychological support for HCWs resulting in severe exhaustion, stress, and higher rates of HCW burnout (Lucero-Prisno III *et al*., 2020*a*, 2020*b*). Figure 1 depicts the various socio-economic factors that contribute to worsening HCW burnout in the country.

The implications of ignoring this calamity include workplace detachment and lower self-efficacy which hinders provision of optimal quality patient care and decreases resilience amongst HCWs (Islam *et al*., 2021*a*). In countries such as Yemen and Lebanon, reports of HCWs leaving the country due to working conditions and stress levels have been reported (Islam *et al*., 2021*a*, 2021*b*). A similar situation is observed in Myanmar during the military coup, where HCWs were arrested, terminated from work, and constantly endangered due to ongoing conflict (Rocha *et al*., 2021).

Amidst these concerns, gender inequality is prevalent in HCWs, with Afghanistan ranked as 171st among 188 countries in the Gender Inequality Index (Najafizada *et al*., 2014; Parray *et al*., 2021). Despite this, an optimistic attitude and continual resilience demonstrated by community care workers, of which half are women, continues to help in the delivery of essential healthcare services (Najafizada *et al*., 2014; Parray *et al*., 2021). The diligence needed to stay motivated during the pandemic emerges from a sense of service and empowerment for female HCWs.

## Efforts and recommendations

A major advancement took place recently when Taliban urged women HCWs to return to work, stating there will be no impediments to them while performing professional duties in attempts to re-establish basic services across the country (Reuters, 2021). In Afghanistan, mental health units are active in six hospitals in order to provide psychosocial support to HCWs and COVID-19 patients (World Health Organization, 2020*b*). This is supported by more than 60 operational hotlines in the country that are providing mental health support and awareness, coupled with referrals to specialized services. Efforts to educate the masses are an essential tool in combating critical situations. Therefore, the World Health Organization (WHO) and the Inter-Agency Standing Committee (IASC) mental health support guidelines were translated into local languages (World Health Organization, 2020*b*). Awareness programs intending to promote self-care and well-being have been developed for different groups, including HCWs and children (World Health Organization, 2020*a*). Moreover, the availability of remote mental health services was advertised on social media, COVID-19 hospitals, and public places, such as mosques and markets (World Health Organization, 2020*a*).

To better advance mental health care in Afghanistan, the allotment of finances and funds for mental health services, and undergraduate training are highly recommended. Devising national health policies and increased research in this area can help navigate the country through this ordeal. Furthermore, trainings to provide suitable psychological first aid and stress management workshops added to undergraduate and graduate-level curriculums could increase community well-being and support. In order to positively cope with the pandemic, several mechanisms and measures must be taken by preparing the HCWs through peer support programs and helping them recognize future challenges through supportive mentoring (Greenberg *et al*., 2020). Furthermore, religion and fatalism also play a role in the context of a Muslim-majority country that values humanitarian efforts as a service to God. Educational programs and institutional training for HCWs could assist them in handling stressful working conditions by employing the use of a meaning-focused approach (Islam *et al*., 2021*a*). On a global scale, the supply of protective personal equipment, vaccines, and medical equipment to support HCWs is vital. International organizations including the WHO need to coordinate with Afghan authorities to negotiate novel ways to provide aid as well as ease restrictions on female HCWs (Essar *et al*., 2021).

## Conclusion

In conclusion, the political instability in Afghanistan is contributing to an impending mental health crisis for HCWs along with the incursion of the COVID-19 pandemic, economic complications, and ongoing civil unrest. As a result, reduced access to mental health has affected the workplace efficacy of HCWs in Afghanistan. Further research is warranted on understanding the impact of conflict on HCWs and help implement vital measures in devising healthcare policies, allocation of finances, and national training focused on mental health recovery.
